# Short- and long-term warming effects of methane may affect the cost-effectiveness of mitigation policies and benefits of low-meat diets

**DOI:** 10.1038/s43016-021-00385-8

**Published:** 2021-12-13

**Authors:** Ignacio Pérez-Domínguez, Agustin del Prado, Klaus Mittenzwei, Jordan Hristov, Stefan Frank, Andrzej Tabeau, Peter Witzke, Petr Havlik, Hans van Meijl, John Lynch, Elke Stehfest, Guillermo Pardo, Jesus Barreiro-Hurle, Jason F. L. Koopman, María José Sanz-Sánchez

**Affiliations:** 1 JRC, Joint Research Centre, European Commission, Seville, Spain; 2 BC3, Basque Centre for Climate Change, Bilbao, Spain; 3 Ikerbasque, Basque Science Foundation, Bilbao, Spain; 4 NIBIO, Norwegian Institute of Bioeconomy Research, Ås, Norway; 5 RURALIS, Institute for Rural and Regional Research, Universitetssenteret Dragvoll, Trondheim, Norway; 6 IIASA, International Institute for Applied Systems Analysis, Laxenburg, Austria; 7 WUR, Wageningen University and Research Centre, The Hague, Netherlands; 8 EuroCARE GmbH, Bonn, Germany; 9 University of Oxford, Oxford, UK; 10 PBL, Netherlands Environmental Assessment Agency, The Hague, Netherlands

## Abstract

Methane’s short atmospheric life has important implications for the design of global climate change mitigation policies in agriculture. Three different agricultural economic models are used to explore how short- and long-term warming effects of methane can affect the cost-effectiveness of mitigation policies and dietary transitions. Results show that the choice of a particular metric for methane’s warming potential is key to determine optimal mitigation options, with metrics based on shorter-term impacts leading to greater overall emission reduction. Also, the promotion of low-meat diets is more effective at reducing greenhouse gas emissions compared to carbon pricing when mitigation policies are based on metrics that reflect methane’s long-term behaviour. A combination of stringent mitigation measures and dietary changes could achieve substantial emission reduction levels, helping reverse the contribution of agriculture to global warming.

Governments around the world have committed to reducing their greenhouse gas (GHG) emissions to limit the global temperature increase to well below 2°C, while pursuing efforts to limit the increase to 1.5 °C^1^. The Paris Agreement^2^ establishes the framework to define countries’ commitments through the elaboration of nationally determined contributions (NDCs). The targets of the Paris Agreement require careful consideration of the mitigation role of the agriculture sector. According to the Synthesis report by the United Nations Framework Convention on Climate Change secretariat based on the aggregate effect of the 161 NDCs communicated by 189 Parties^3^, 74% of the countries that have communicated their NDCs include GHG reduction in the agricultural sector and 80% and 77% of the countries cover methane (CH_4_) and nitrous oxide (N_2_O) emissions in their NDCs, respectively. Recent updates indicate that 57% and 62% of countries submitting NDCs cover CH_4_ and N_2_O emissions, respectively^4^. Mitigation targets for non-CO_2_ GHG emissions from agriculture are mostly conditional for developed countries. However, agricultural emission reduction policies remain a long way from achieving the substantial reductions that are suggested by modelled scenarios compatible with limiting warming to 1.5–2 °C^5^. In addition, there are ongoing discussions around the role of short-lived GHGs such as CH_4_, and associated metrics, with particularly relevant implications for agriculture emission reduction policies and how the contribution of the sector to climate change mitigation is perceived.

GHG emission metrics pursue the goal of comparing the global warming contributions of different climate gases in a transparent and understandable way, without compromising climate scientific knowledge. National GHG inventories, which follow common methodological guidance provided by the Intergovernmental Panel on Climate Change (IPCC)^6,7^ are used to report GHG emissions and removals towards national binding commitments (that is, Kyoto Protocol quantified emission limitation and reduction objectives and Paris Agreement NDCs), and therefore also as accounting tools to check compliance against such commitments.

Non-CO_2_ GHG emissions are commonly reported as ‘CO_2_-equivalents’ (CO_2_e) and calculated using the 100 yr global warming potential (GWP_100_)^6–8^ NDCs in which nations set out their emission reduction targets, and economic costing tools valuing different emissions (or mitigations thereof) are largely built on this approach. As a metric that provides a single per-emission weighting of each gas, the GWP_100_ fails to capture how the relative impacts of different gases change over time. Due to its short atmospheric lifetime, the impacts of CH_4_ emissions rapidly decline after a few decades. Meanwhile, due to its long lifetime, each CO_2_ emission exerts a relatively stable impact on global temperature into the long term. The relative valuation of CH_4_ to CO_2_ is thus highly sensitive to the metric used, particularly the metric’s time horizon^9–11^.

Proposals to account for this effect include adding supplementary information to NDCs about the emissions levels and/or separate targets for individual GHGs (for example, New Zealand has a separate target to reduce biogenic CH_4_ emissions), and/or reporting aggregated emissions using different metrics, such as shifting among conventional GWPs with different time horizons, for example, GWP20 (ref. ^12^), or using alternative metric approaches, for example, GWP* (refs. ^13,14^). While this debate on the usefulness of alternative metrics is still ongoing in the scientific literature, the fact that CH_4_, as a short-lived gas, has distinct impacts whether viewed over the shorter or longer term is well established.

As CH_4_ is responsible for a large proportion of global GHG emissions, changes to the valuation of CH_4_ relative to CO_2_ can strongly affect how much the agricultural sector is forced by policy decision-makers to reduce its GHG emissions and responds to ‘carbon pricing’. In addition, the contrasting lifetimes of the two gases result in distinct warming dynamics, which should be kept in mind when considering the nature of agriculture’s contribution to global warming. Therefore, these considerations could have substantial implications for how agroeconomic policies are designed and evaluated as well as what policy recommendations are put forward. In this article we explore the impacts of acknowledging the distinct differences between short-lived and long-lived climate gases in mitigation frameworks.

## Agriculture’s contribution to climate change mitigation efforts

An ensemble of large-scale economic land-use models was used to quantify the cost-effective contribution of agriculture to mitigating climate change under different valuations of CH_4_ based on a similar set of counterfactual scenarios as in ref. ^15^). The three economic models (CAPRI, GLOBIOM and MAGNET) provide detailed representations of the agricultural sector, cross-sectoral linkages through factor markets and substitution effects and GHG emissions by agricultural production activity. Our focus was on the reduction of agricultural emissions over time and their effective contribution to climate change, differentiating between sources (for example, ruminant, dairy and rice production) and world producing regions^16^.

We analysed how mitigation policies (focusing either on the short- or long-term effects) affect emission reductions and the consequences for the agricultural sector by way of two mitigation options. First, a global carbon price path on the supply side, inducing both the implementation of technical mitigation options to reduce emission intensity and affecting production (structural changes and production levels) as described in ref. ^15^. Second, a change towards lower consumption of animal-protein-based diets on the demand side (see Table 1 for an overview of the scenarios analysed). ‘Carbon pricing’ is widely considered an efficient means to achieve the ambitions set out in the Paris Agreement^17–20^. Monitoring of CH_4_ emissions from agriculture is not an easy task due to their biological nature, diverse land-use techniques and widely different farm management practices^21–23^, and therefore direct emission taxation may be problematic. Independent of the practical challenges, carbon pricing has been applied in agricultural economic models as a means to identify the cost-effective potential, or as an approximation of other mitigation policies^15,24^ Moreover, the economic models applied considered a global mitigation cost curve as the estimate of the aggregated mitigation potential and costs of specific mitigation technologies^25,26^.

In this study we explored alternative CH_4_ valuations based on the discussions that have arisen over implications of the short-lived character of CH_4_ by means of scenarios combining two different carbon prices (US$150 and US$500 t^−1^) on non-CO_2_ agricultural emissions and a low-animal-protein diet (Table 1). Conventionally, the impact of CH_4_ is made comparable to CO_2_ via the GWP_100_, describing the integral of the induced radiative forcing over time (100 yr) compared to that of CO_2_. By using the integral, both short-term strong warming and its fast decay are included. However, when the ambition is to reduce warming in the next few decades, a shorter time horizon might be applied in comparing the effects of CO_2_ and CH_4_. Moreover, if one wants to stress that CH_4_ only has little effect on warming in the long term, a longer perspective or end-point temperature might be appropriate. The GWP* method^13,14^ stresses that the short-term effect of CH_4_ is four times higher than in the conventional GWP_100_ method, but is only 0.25 of the conventional GWP_100_ (3.75 of the initial 4 is reversed) in the long term (equation (1)). To reflect ‘short-term’, ‘conventional’ or ‘long-term’ perspectives, we applied all three in the pricing schemes. With the GWP_100_ of CH_4_ being 25, this resulted in methane equivalence factors (MEFs) of 100, 25 and 6.25, respectively. The short- and long-term factors, 100 and 6.25, are in fact close to the 20 yr global warming potential (GWP_20_), 84, and to the 100-year global temperature change potential (GTP_100_), 4 (IPCC 5th Assessment Report (AR5)), and these two metrics, GWP_20_ and GTP_100_, have also been recommended as alternative metrics for life-cycle assessment to consider the shorter and longer term^27^.

To analyse effective mitigation by the agricultural sector in these scenarios, we reported both emissions and added warming. Added warming from CO_2_ was assessed by applying the so-called transient climate response to cumulated carbon emissions (TCRE)^28^. This measure can be applied across all GHGs when using appropriate CO_2_-equivalence emissions. GWP* was designed so that cumulative GHG emissions are correlated with added warming^13^, similar to what GWP_100_ does for CO_2_, N_2_O and other long-lived gases, but not for short-lived gases such as CH_4_ (refs. ^13,14^) (Methods).

In addition to mitigation efforts targeting the supply side, reduced consumption of animal-protein-based diets has also been identified as a promising strategy to curb GHG emissions from the agriculture and global food systems^29–31^. Following ref. ^15^, we assumed a threshold on animal product calories of 430 kcal per capita per day (ruminant, non-ruminant and dairy production). This target excluded food waste and is assumed to be achieved by 2070, such that calorie consumption decreased linearly from current levels. No increase in consumption of vegetable calories was assumed, but it might occur endogenously. Model results showed a decrease in average global calorie consumption by at most 3.8% in 2070. This difference could be replaced by a larger consumption of legumes, increasing global agricultural area by up to 1.5% in 2070 compared with the baseline in that year, or simply left unchanged as a measure to reduce overweight and obesity.

Table 1 shows the mitigation and dietary shift scenarios analysed by the three economic models.

## Long-term methane emissions under business as usual

**Fig. 1:**
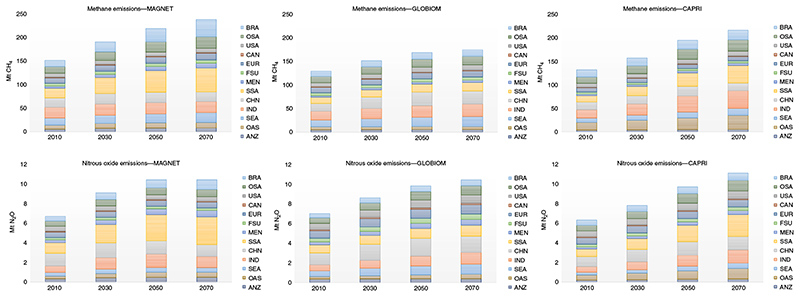
Baseline methane emissions. Regional totals for scenarios by baseline year and model (Mt CH_4_). ANZ, Australia and New Zealand; OAS, other Asia; SEA, Southeast Asia; IND, India; CHN, China; SSA, sub-Saharan Africa; MEN, Middle East, North Africa and Turkey; FSU, former Soviet Union; EUR, Europe; CAN, Canada; USA, United States of America; OSA, other South, Central America and Caribbean (including Mexico); BRA, Brazil.

Our business-as-usual scenario (BASE) with no GHG mitigation policy corresponds to the Shared Socioeconomic Pathway 2 (SSP2), a ‘middle-of-the-road’ scenario which depicts a future of global development where developing countries achieve important economic growth^32,33^. With these assumptions, global agricultural CH_4_ emissions are expected to increase by over 50% between 2010 and 2070, reaching 170–240 Mt yr^−1^ in 2070, depending on the model. This increase comes at a decreasing rate (Fig. 1), reflecting a certain convergence of red meat and dairy consumption worldwide. In fact, while developed economies show a stable trend, developing countries continue increasing their intake of animal protein from very low levels. Most of the increase in CH_4_ emissions is to be attributed to higher productivity per animal in ruminant production, with cattle numbers slightly increasing (beef herds) or even decreasing (dairy herds).

Methane emission projections are very different from a regional perspective, which needs to be considered when mapping global mitigation initiatives into national policies. By 2010, about 57% of total agricultural CH_4_ emissions were coming from India, China, Brazil, sub-Saharan Africa and Southeast Asia. By 2050 and 2070, these regions are expected to increase their share to about 62%. In CAPRI and MAGNET, sub-Saharan Africa and India are expected to remain as the largest CH_4_ emitters, with about 40–50% of total CH_4_ emissions in all models. China, in turn, is characterized by stable or slightly decreasing emissions, depending on the model projection. GLOBIOM, in turn, projects a larger emission share for China for 2050 and 2070.

**Fig. 2:**
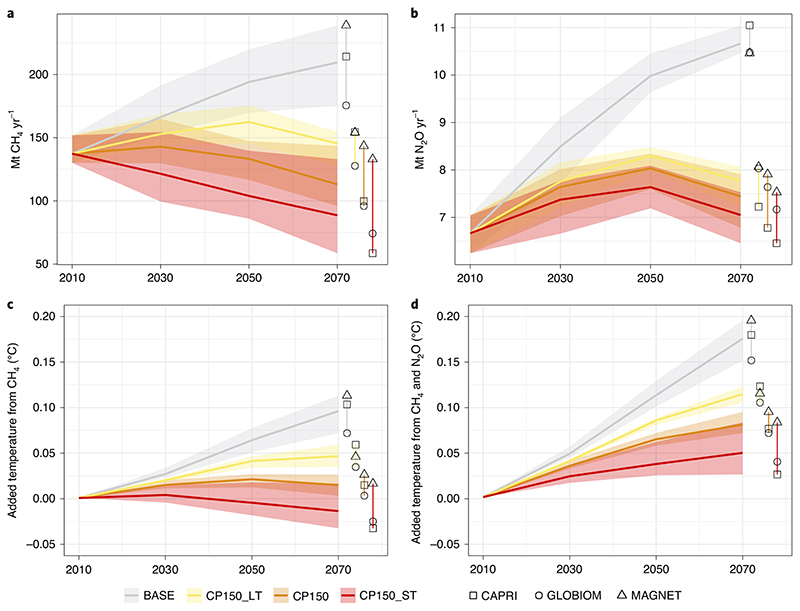
Methane and nitrous oxide emissions for the baseline and US$150 t^−1^ carbon price scenarios. **a**–**d**, World totals by year and model: annual Mt CH_4_ (**a**); annual Mt N_2_O (**b**); added warming for CH_4_ emissions (**c**); and added warming for total non-CO_2_ emissions (**d**). The shading is the range (distribution across models for respective scenario) compared to the average (thick middle line).

We present induced warming from both gases (as described above) relative to 2010. Under the baseline, increasing CH_4_ emissions alone induce a warming of about 0.1 °C, and about 0.175 °C together with N_2_O (grey shaded areas in Fig. 2c,d).

## Emission mitigation

### Carbon pricing

The implementation of a carbon price of US$150 t^−1^ (CP150) to the agricultural sector based on GWP_100_ yields an average reduction of 12%, 28% and 40% in CH_4_ emissions in 2030, 2050 and 2070, respectively, compared to the baseline in those same years (Fig. 2a). Similarly, the average impact CP500 constitutes a reduction of 23%, 40% and 53% in CH_4_ emissions for the three projections, respectively (Supplementary Fig. 2a). The impact of these CH_4_ emissions for additional global warming is positive for CP150 (that is, causes warming relative to 2010; Fig. 2c), but turns negative for CP500 in 2070 (that is, causes cooling relative to 2010; Supplementary Fig. 2). High carbon pricing thus leads to a substantial reduction in CH_4_ emissions, partially reversing some of the warming CH_4_ emissions previously caused, as CH_4_ concentrations (and subsequently their contribution to warming) will fall if emission rates sufficiently decline. Despite that contribution, the warming effect of total non-CO_2_ emissions from agriculture remains positive (+0.05 °C; Fig. 2d).

We further investigate the effects of a carbon price scheme that focuses on either the short-term or long-term temperature impact of CH_4_ emissions as explained above. For this, we model carbon pricing based on the short-term effects (CP150_ST and CP500_ST) and on the long-term effects (CP150_LT and CP500_LT) derived from the original carbon price scenarios. A carbon price scheme focusing on the short-term temperature effect of CH_4_ considerably reduces CH_4_ emissions (Fig. 2a) and turns the implied warming effect of this declining emission path negative (Fig. 2c). CP150_ST yields CH_4_ emission reductions of about 41% and CP500-ST 60% in 2070. However, emission reductions are considerably less than proportional to the carbon price increase. In fact, at high carbon price levels technological options for mitigation are exhausted and agricultural systems become very constrained, facing severe income losses (Table 2). The results for CP500_ST and CP500-LT can be found in the Supplementary Information. Figure 2b shows the indirect impact of the GWP_100_-based carbon price scheme on N_2_O emissions and Fig. 2d the aggregated impact on added temperature for both CH_4_ and N_2_O.

The difference in model responses to the carbon pricing schemes comes from the different response of producers and consumers to the increased carbon pricing scheme which results from applying the different metrics. For instance, compared to CAPRI and GLOBIOM, the MAGNET model assumes that consumers are more willing to pay the higher prices for meat that result from the increased carbon prices (and thus are willing to spend a larger fraction of their total income on food). Moreover, in MAGNET part of the sales of the meat sector are going to non-food sectors such as the chemical sector (for example, fats) which can easily pay the increased price. Furthermore, the mitigation options available to the sector are limited after the initial reductions compared to the baseline due to a steeper marginal abatement cost curve for initial carbon prices in MAGNET. The willingness of all consumers to pay more for ruminants is also reflected in the higher producer prices for ruminants in Supplementary Fig. 5.

### Carbon pricing and low-animal-protein diets

**Fig. 3:**
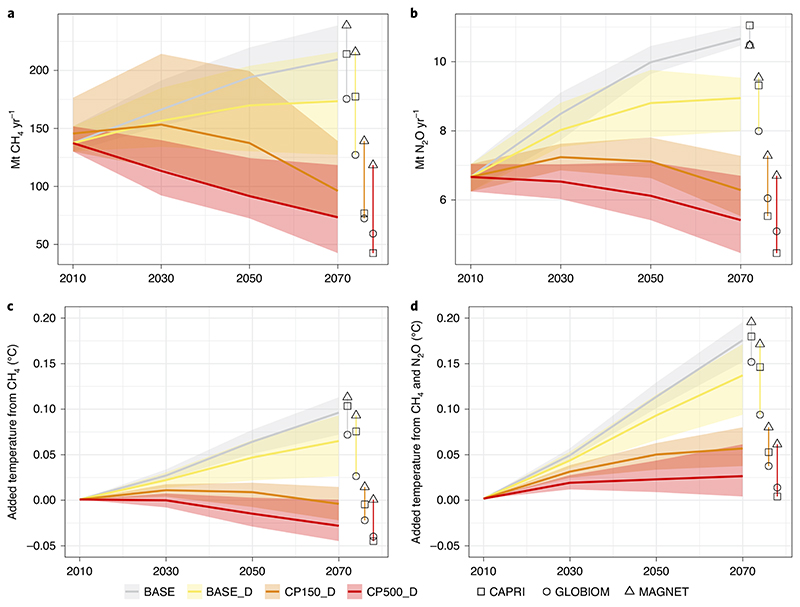
Methane and nitrous oxide emissions for the baseline and scenarios including dietary shifts with and without carbon pricing. **a**–**d**, World totals by year and model: annual Mt CH_4_ (**a**); annual Mt N_2_O (**b**); added warming for CH_4_ emissions (**c**); and added warming for total non-CO_2_ emissions (**d**). The shading is the range (distribution across models for respective scenario) compared to the average (thick middle line).

While the simulated dietary shifts lead to further emission reduction on top of carbon pricing, dietary shifts alone have a lower impact than carbon pricing on emission reduction—at least for the given assumptions (Fig. 3c). When dietary shifts are combined with carbon pricing, induced warming from CH_4_ compared to 2010 turns negative for both carbon prices.

Adding dietary shifts to carbon pricing that focuses on either the short-term or the long-term effect of CH_4_ emissions does not change the main results compared to a situation without dietary shifts. However, the magnitude of the impact is different. The additional impact of dietary shifts on reducing induced warming becomes larger (smaller) if the carbon price is based on the long-term (short-term) effect of CH_4_ emissions. Moreover, that effect decreases with the carbon price level consistently across all scenarios: the larger the reduction in warming due to carbon pricing, the less effect dietary shifts will have. This is because higher carbon prices lead to more technical emission reduction measures, reducing the emission intensity of foods and hence reducing the magnitude of the effects of dietary shifts. When mitigation efforts are based on the long-term effects of CH_4_, carbon pricing becomes a less powerful mitigation tool relative to dietary shifts.

The opposite holds when carbon pricing focuses on the short-term effect of CH_4_. In this case, CH_4_ is priced stronger and the additional effect of dietary shifts decreases (Table 2). In all scenarios, in absolute terms, carbon pricing remains more important for mitigation than a dietary shift.

### Impact on global agriculture

Carbon pricing and dietary changes lead to a contraction of agricultural production (Table 2). Dietary shifts have a larger impact on production than carbon pricing. In the absence of carbon pricing, a dietary shift leads to a 13% reduction in 2070 compared to the baseline. Adding a carbon price has minor additional impact. When dietary changes are considered, production drops between 15% and 18% depending on the carbon pricing regime and the carbon price level. However, if no dietary shifts are considered, production only decreases between 2% and 8% depending on the carbon price regime and level. The reason for this result is twofold. First, carbon pricing allows, and incentivizes, farmers to implement mitigation options without necessarily reducing production. Second, farmers can pass some of the costs on to consumers to better maintain profitability in production. Table 2 indicates that the prices farmers receive at the farm gate increase in the presence of carbon pricing. In the case of a dietary shift, demand simply decreases and producer prices fall. In fact, in the absence of carbon pricing, producer prices fall by 16%. They still decrease up to 11% in the long term with the lower carbon tax (CP150_LT_D), but the pure price effect from the dietary shifts is mitigated by the carbon tax. Carbon taxes drive up the production costs, which is translated into higher producer prices. This effect is visible in CP500_ST_D, where the carbon tax is highest and price impacts vary from a 2% decline (MEF-LT) to a 26% increase (MEF-ST).

The carbon pricing regime seems to have a limited effect on overall agricultural production, but a stronger effect on producer prices. Carbon pricing regimes that focus either on the short-term or the long-term warming impact of CH_4_ emissions result in a 1 percentage point deviation of production compared to carbon pricing using the conventional GWP_100_. Overall production decreases 4% under a carbon pricing regime based on the short-term warming impact of CH_4_, while the reduction is 2–3% under a GWP_100_ pricing regime with a carbon price of US$150 t^−1^. A decomposition of the production effects reveals that carbon pricing leads to a decline in both crop, non-ruminant and ruminant production under all carbon pricing regimes and for all carbon price levels. As a major source of CH_4_ emissions, ruminant production experiences the largest decrease of the three types of production. At the same time, it is heavily affected by the choice of the carbon pricing scheme. About 40% of the decrease in ruminant production is avoided if a carbon pricing regime based on the long-term warming impact of CH_4_ is employed rather than conventional GWP_100_ with a carbon price of US$150 t^−1^. The carbon pricing regime plays a much smaller role if a dietary shift causes the reduction in ruminant production. In this case, only one-tenth of the drop in ruminant production is reversed.

Producer prices show larger impacts, in particular when the carbon pricing regime focuses on the short-term warming impact of CH_4_. For a carbon price of US$500 t^−1^, prices increase by 24% under conventional CP500, but 51% under CP500-ST (Table 2). Moreover, the uncertainty expressed through variation in results across models around those price changes is larger for higher carbon prices and short-term focus (Supplementary Fig. 4).

**Fig. 4:**
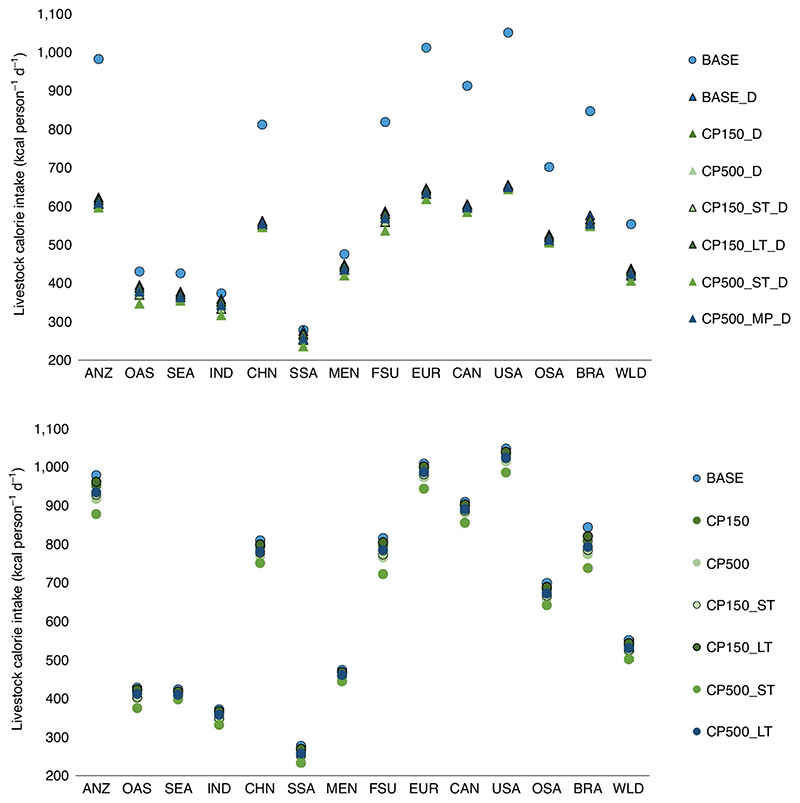
Change in livestock calorie consumption in the year 2050. **a**,**b** Regional totals for scenarios including a dietary shift (**a**) and scenarios with no dietary shift (**b**). DRY, milk; RUM, ruminant meats; NRM, non-ruminant meats; RIC, paddy rice; CER, cereals; OCR, other crops.

Since the change in diets is also partially taking place through changes in the composition of food supply and reduction in production volumes, we finally compare in Fig. 4 the impact on livestock calorie consumption of carbon pricing with and without dietary shifts. While global livestock per-capita calorie consumption is reduced in the dietary shift scenarios by about 4–18% in 2030, 13–31% in 2050 and 23–36% in 2070, depending on the model, the reduction is much lower when only considering carbon pricing (around 9% achieved by the CP500_ST scenario). This calorie reduction takes place only in emerging economies with high meat consumption (for example, China, former Soviet Union, Brazil) and developed countries (European Union, United States, Canada, Australia and New Zealand).

## Discussion

The transiency of CH_4_ emissions is key for determining cost-effective climate change mitigation options in the agricultural sector and assessing their impact in a rigorous manner. We show how different valuations of CH_4_ relative to CO_2_ impact the choice of mitigation policies in agriculture and, consequently, affect the sector’s contribution to further global warming.

While a number of earlier studies, such as Smith et al.^34^, Reisinger et al.^35^, van den Berg et al.^36^ and Strefler et al.^37^, have explored the implications of different CH_4_ valuations on emission abatement, only Reisinger et al.^35^ includes a specific breakdown of agricultural impacts. In this paper, we go further, using a multimodel comparison of updated agricultural economic models, including the independent impacts of dietary shifts (that is, shifts to low-animal-protein diets), and reporting the global warming contribution of our agricultural emission scenarios.

Our research underlines the fact that emission accounting metrics have an impact on climate mitigation policy options. This question deserves further analysis within the IPCC AR6 process and will certainly become more prominent as the share of agricultural emissions post-2030 will increase as widespread decarbonisation will rapidly start reducing emissions from other sectors that currently dominate. Conventionally, the impact of a certain sector on climate is evaluated though its annual GHG emissions, typically aggregated and reported in GWP_100_. However, due to the short-lived character of CH_4_, (cumulative) GWP_100_ CO_2_e emissions do not necessarily correctly reflect implied warming, especially not under stringent mitigation scenarios. We therefore present here explicitly the warming induced by agricultural CH_4_ and N_2_O emissions.

Decreasing CH_4_ emissions from agriculture can have a negative warming effect, as revealed when using the GWP* metric (Fig. 2c). In this respect, decreasing CH_4_ emission rates have, in terms of overall climate impact relative to current temperatures, the same effect as CO_2_ uptake or carbon capture and storage technologies. This may allow for some leeway in the design of climate policy packages and consideration of whether some emissions may ultimately be considered compatible with climate targets. However, this effect is scenario dependent and does not necessarily apply to agriculture overall when considering all GHGs. Our analysis shows that total agricultural emissions will contribute to further global warming irrespective of the carbon pricing regime and carbon price level. Compared to Frank et al.^15^, the global warming impact remains unchanged until 2050 and starts decreasing while getting close to 2070, mainly due to a regional convergence of world animal protein consumption and technology adoption induced by carbon pricing. These results are linked to agricultural emission pathways based on medium- and long-term projections of agricultural markets.

Consistent with earlier studies on the contribution of agriculture to stringent climate mitigation efforts^15,38^ we find that comparable carbon pricing would reduce agricultural CH_4_ and N_2_O emissions by up to 58% and 53%, respectively, compared to the baseline in 2070 and reduce aggregate warming above 2010 levels to zero in 2070 (from 0.17 °C in the baseline). Focusing specifically on the short-term effect of CH_4_ will lead to even larger reductions in CH_4_ emissions, but will come with more severe impacts in the agricultural system in terms of prices and production indices. The impact of low-animal-protein diets as a mitigation option strongly depends on the context in which this trend is occurring. Reductions in meat consumption and production will considerably contribute to climate stabilization and become a powerful mitigation technology if carbon pricing is moderate (Fig. 3c,d).

Emission mitigation policies could have an ambiguous effect on livestock production if society gives more value to the long-term effect of CH_4_. On the one hand, carbon pricing based on the long-term warming impact of CH_4_ relieves pressure to reduce cattle herds. On the other, that pricing regime also highlights the large immediate benefits of reducing CH_4_, and dietary change has a greater effect in this case, where lower carbon pricing has resulted in fewer technical measures to reduce CH_4_ emissions. Moreover, while carbon pricing leaves farmers with the option to implement less-emitting technologies, a dietary shift simply means fewer cows.

Our results highlight—beyond the sheer emission and warming effects—the differential impact of various carbon pricing levels and dietary shifts on the agricultural sector. Carbon pricing has in general the largest effect on emissions, but with increasing carbon price levels, the negative economic impacts on the agricultural sector in terms of lower production continue to increase, while further emission reductions are relatively small. This reflects a situation where the technical abatement options are fully applied and further reduction comes from price-induced reduction in consumption^38^. Consequently, incentives for agricultural mitigation should exploit all technical abatement options that are feasible but also carefully address regionally specific consumption effects.

In this context, we note that although multigas mitigation policies are expected to prove more cost effective than CO_2_-only approaches^39^, the distribution of costs across different sectors can be uneven, with, for example, higher CH_4_ valuations increasing costs for agriculture and predominantly benefiting the energy sector^35^. Therefore, while this study focused on agriculture alone, it would be useful for further work to explore interactions with other sectors, and policy formulation should be mindful of distributional issues that may arise from different emission pricing options.

Our results come with some limitations. First, we apply a comparative-static modelling framework to a dynamic decision problem. Second, our model exercise disregards the costs of monitoring emissions and inducing dietary shifts. Refraining from these transaction costs, our analysis potentially overestimates the efficiency of the investigated mitigation options. More research is needed to develop and analyse how a switch to metrics (or simply modelling approaches that do not require metrics) that better reflect the warming potential of different climate pollutants can be implemented in practice and whether transaction costs will reduce the efficiency of these mitigation options.

## Methods

### Description of the models

The CAPRI (Common Agricultural Policy Regionalised Impact) modelling system is an economic large-scale, comparative-static, partial equilibrium model focusing on agriculture and the primary processing sectors. CAPRI comprises two interacting modules, linking a set of mathematical programming models of EU regional agricultural supply to a spatial multicommodity model for global agrifood markets. The regional supply models depict a profit-maximizing behaviour of representative farms in the European Union and candidate countries, taking into account constraints related to land availability, nutrient balances for cropping and animal activities and policy restrictions^40^. The market module consists of a spatial, non-stochastic global multicommodity model for about 60 primary and processed agricultural products, covering 77 countries in 40 trading blocks. Bilateral trade flows and attached prices are modelled based on the Armington assumption of quality differentiation^41^. The behavioural functions in the market model represent supply and demand for primary agricultural and processed commodities (including human and feed consumption, biofuel use, import demand from multilateral trade relations), balancing constraints and agricultural market policy instruments (that is, import tariffs, tariff rate quotas, producer and consumer support estimates, and so on). Depending on scenarios, behavioural functions are shifted (for example, to reflect productivity shocks or preference shifts) and the model solves for the new market equilibrium.

With regard to GHG accounting, CAPRI calculates EU agricultural GHG emissions for the most important N_2_O and CH_4_ emission sources based on the inputs and outputs of agricultural production activities, following to a large extent the 2006 IPCC guidelines. It also takes into account detailed technical and management-based GHG mitigation options for EU agriculture. GHG emissions for the rest of the world are estimated on a commodity basis in the market model^42,43^ GHG mitigation in non-European countries is represented by a change in emission factors and a matching change in output prices to reflect the increase in cost, derived from mitigation cost functions from the literature^25^. In terms of the database the European data are mostly sourced from Eurostat, while the international data are mostly from the Food and Agriculture Organization, for both model parts supplemented by topic-related sources.

The Global Biosphere Management Model (GLOBIOM)^44^ is a partial equilibrium model that covers the global agricultural and forestry sectors, including the bioenergy sector. Commodity markets and international trade are represented at the level of 35 economic regions in this study. Prices are endogenously determined at the regional level to establish market equilibrium to reconcile demand, domestic supply and international trade. The spatial resolution of the supply side relies on the concept of simulation units, which are aggregates of 5–30 arcmin pixels belonging to the same altitude, slope and soil class, and the same country^45^. For crops, livestock and forest products, spatially explicit Leontief production functions covering alternative production systems are parameterized using biophysical models such as EPIC (Environmental Policy Integrated Model)^46^, G4M (Global Forest Model)^47^ or the RUMINANT model^48^. For the present study, the supply-side spatial resolution was aggregated to 2° (about 200 × 200 km at the equator). Land and other resources are allocated to the different production and processing activities to maximize a social welfare function which consists of the sum of producer and consumer surplus. The model includes six landcover types: cropland, grassland, short rotation plantations, managed forests, unmanaged forests and other natural vegetation land. Depending on the relative profitability of the production activities of primary products, by-products and final products, the model can switch from one landcover type to another. Spatially explicit land conversion over the simulation period is endogenously determined within the available land resources and conversion costs that are taken into account in the producer optimization behaviour. Land conversion possibilities are further restricted through biophysical land suitability and production potentials, and through a matrix of potential landcover transitions. GLOBIOM covers major GHG emissions from agricultural production, forestry and other land use including CO_2_ emissions from above- and belowground biomass changes, N_2_O from the application of synthetic fertilizer and manure to soils, N_2_O from manure dropped on pastures, CH_4_ from rice cultivation, N_2_O and CH_4_ from manure management, and CH_4_ from enteric fermentation. For this study, only results for non-CO_2_ emissions were reported.

GLOBIOM explicitly covers different mitigation options for the agricultural sector. Technical mitigation options such as anaerobic digesters, livestock feed supplements, nitrogen inhibitors, and so on, are based on ref. ^49^. Structural adjustments are represented through a comprehensive set of crop and livestock management systems parameterized using biophysical models, that is, transition in management systems, reallocation of production within and across regions^44^ and consumers’ response to market signals^50^. Detailed information on the parameterization of the different mitigation options for the agricultural sector is presented in ref. ^38^. For more information on the general model structure we refer to refs. ^44,51^.

The Modular Applied GeNeral Equilibrium Tool (MAGNET) model is a multiregional, multisectoral, applied general equilibrium model based on neoclassical microeconomic theory^52,53^. It is an extended version of the standard GTAP model^54^. The core of MAGNET is an input–output model, which links industries in value-added chains from primary goods, over continuously higher stages of intermediate processing, to the final assembly of goods and services for consumption. Primary production factors are employed within each economic region, and hence returns to land and capital are endogenously determined at equilibrium, that is, the aggregate supply of each factor equals its demand. On the consumption side, the regional household is assumed to distribute income across savings and (government and private) consumption expenditures according to fixed budget shares. Private consumption expenditures are allocated across commodities according to a non-homothetic constant difference of elasticity expenditure function, and government consumption according to Cobb–the Douglas expenditure function.

The MAGNET model, in comparison to GTAP, uses a more general multilevel sector-specific nested constant elasticity of substitution production function, allowing for substitution between primary production factors (land, labour, capital and natural resources) and intermediate production factors, and for substitution between different intermediate input components (for example, energy sources and animal feed components). MAGNET includes an improved treatment of agricultural sectors, examples include: various imperfectly substitutable types of land; the land-use allocation structure; a land-supply function; substitution between various animal-feed components^53,55^, agricultural policy (such as production quotas and different land-related payments) and biofuel policy (capital-energy substitution, fossil fuel–biofuel substitution^56^). On the consumption side, a dynamic constant difference of elasticity expenditure function is implemented which allows for changes in income elasticities when purchasing-power-parity-corrected real gross domestic product per capita changes. Segmentation and imperfect mobility between agriculture and non-agriculture labour and capital are introduced in the modelling of factors markets.

MAGNET calculates absolute non-CO_2_ GHG emissions resulting from agricultural production which depends on demand (gross domestic product, population, diet and bioenergy use) and productivity. Emission intensities (that is, emissions per unit of production) are determined through model-specific emission factors. In addition, emission intensities change in the SSP2 baseline scenario due to the following assumptions on technological improvements: (1) nitrogen fertilizer substitution with labour, capital and land; (2) yield increases due to exogenous technological improvements (adopted from IMAGE) and endogenous improvements due to substitution of land with fertilizer and land–fertilizer bundle with labour and capital; and (3) exogenous feed use efficiency by livestock (adopted from the IMAGE model^57^) and endogenous substitution between different feed components.

In MAGNET most of the CH_4_ emissions scale with the output of the agricultural sector and so taxing the emissions is equivalent to a tax on output. This is also the case with N_2_O emissions from the livestock sectors. For the crop sectors, however, N_2_O emissions come mostly from the application of synthetic fertilizer which can be substituted for land. If the land price rises (declines) the crop sectors will have an increased incentive to apply more (less) fertilizer and use relatively less (more) land. Marginal abatement cost curves are exogenously implemented based on calculations per sector and region from the IMAGE model. For every period the CO_2_ price would correspond to a particular level of emission abatement by technical means (that is, farmers would have an incentive to invest in abatement technology) which would be reflected in a reduction of the emission coefficient for a particular agricultural sector. The additional cost of this abatement would be added to the effective carbon price applied to the sector.

### Scenario construction

The scenarios considered are counterfactual to a long-term ‘business as usual’ projection of agricultural commodity markets and are presented to provide a more comprehensive perspective of how global mitigation policies and dietary policies could contribute to the temperature target set by the Paris Agreement under GWP_100_ and GWP* metrics. Focus is on the reduction of agricultural CH_4_ emissions over time and their effective contribution to climate change, differentiating between sources (for example, ruminant, dairy and rice production) and world producing regions.

To analyse the economic impact of global climate mitigation policies we use a global carbon price path as a proxy for a global mitigation effort^15^. The impacts of this global carbon price on CH_4_ emissions depends on the emission metric applied, and will differ from the standard GWP_100_ if the GWP* metric is applied due to the introduction of time dynamics in its calculation. This is achieved through the following equation, presented as in the simplified rearrangement from ref. ^11^:

(1) $$E_{{\text{CO}}_{2}\text{-w}{\text{.eq.}}_{({\text{CH}}_{4})}}={\text{GWP}}_{100}\times \left(4\times E_{{\text{CH}}_{4}(t)}-3.75\times E_{{\text{CH}}_{4}(t-20)}\right)$$

where ‘CO_2_-warming-equivalent’ emissions (ECO_2-w.eq._) have a large initial effect at the time of release (four times the conventional GWP_100_ valuation), but much of this (3.75 times the conventional GWP_100_ valuation) is considered reversed 20 yr later. Consequently, the reported CO_2_e valuation of CH_4_ emissions can be higher for those sources where emissions have increased over time and can be negative for those cases where emissions have decreased.

Added temperature from CH_4_ emissions is computed in equation (2):

(2) $${\text{AW}}_{{\text{CH}}_{4}(t)}=E_{{\text{CO}}_{2}\text{-w}{\text{.eq.}}_{\left({\text{CH}}_{4}\right)}}\times \text{TCRE}$$

where AW_CH_4_ (t)_ is the added warming (that is, temperature increase or decrease) in year *t* relative to year *t* − 20. For TCRE (that is, the transient climate response to cumulated carbon emissions)^28^, we use the observationally constrained best estimate of 1.8 °C per TtC^13^, which is converts into 0.49 °C per TtCO_2_.

‘Carbon pricing’ is widely acknowledged as an efficient means to achieve the ambitions set out in the Paris Agreement^17–20^; however, it requires a transparent, predictable and practicable monitoring system that reports emissions at their source. Emissions from agriculture differ from emissions from standardized industrial processes due to their biological nature, diverse land-use techniques and different farm-management practices, leading to large variations in emission intensities for identical products^21–23^. In addition, the spatial dispersion of farming renders the accurate monitoring of agricultural emissions at their source almost impossible. Carbon prices have therefore been applied in agricultural economic models as an approximation of other policies that incentivize farmers to implement mitigation options (or penalizes them for not adopting them), while the transaction costs caused by those policies have been neglected^15,24^.

Notwithstanding the fact that monitoring emissions in agriculture involves high transaction costs, the change in the GHG accounting metric to GWP* makes the implementation of a carbon price in agriculture more complicated because the warming impact of CH_4_ emissions needs to be based on two points in time, 20 yr apart. In effect, the full impact of emissions is delayed by 20 yr, raising concerns about who can be made responsible for paying the carbon price. GWP_100_ accounts for emissions only in the year in which they originate. On the contrary, GWP* accounting requires the year emissions occur, and 20 yr before, to reflect that short-lived CH_4_ is rapidly destroyed in the atmosphere via natural processes. A carbon price that requires a time span of 20 yr to be calculated is difficult to administer. Neither the farmer nor the firm responsible for the emissions will necessarily be the same after 20 yr. (This 20-yr time span is suggested in ref. ^13^, which has the effect of reducing the volatility in CO_2_-w.eq. emissions and improving the correspondence with temperature response.)

For this paper, we consider two options for the computation of the carbon price: a ‘short term’ (MEF-ST) and a ‘long term’ (MEF-LT) one, separating out the two components of the GWP* equation.

The MEF-ST option focuses on the strong impact of changing CH_4_ emission rates, computing the carbon price based on the initial valuation at the point an emission occurs, and neglecting the subsequent reversal of most of the emission’s impact in the years ahead. Consequently, the resulting carbon price will be four times higher than a GWP_100_-based carbon price, leading to strong incentives to mitigate CH_4_ emissions. The MEF-ST option could reflect motives of decision-makers that prioritize reducing overall GHG emissions fast, that is, almost independent from any metric.

The MEF-LT option aims at implementing a GWP*-based carbon price assuming that the CH_4_ dynamics are perfectly understood by economic actors and credibly enforced by regulators. Since we apply static models to a dynamic planning problem, we simplify the planning problem by assuming Hotelling’s rule^58^. This rule states that the optimal price path of a non-renewable, durable resource follows the discount rate. We assume that every CO_2_ emission causes the same damage, and, consequently, no tipping points are considered. In our case, that resource would be the CO_2_-absorbing capacity of the atmosphere. With constant carbon prices in real terms, the carbon price will be understood as a regime that charges emissions four times the GWP_100_-based carbon price the year they accrue, but rewards a rebate of 3.75 times that price 20 yr later. The net effect is a price of (4 – 3.75 =) 25% of the GWP_100_-based carbon price. The MEF-LT option thus reflects the fact that ‘after 20 years much of the warming caused by an individual CH_4_ emitter is automatically reserved’^11^. In our models, the MEF-LT carbon price is implemented with its net effect in the year emissions occur.

The two options above only regard the pricing of CH_4_. The computation of the carbon price for CO_2_ and N_2_O is not affected by GWP* and follows GWP_100_ in both options. Although N_2_O is less durable than CO_2_, it is not considered a short-lived climate gas like CH_4_. These globally uniform carbon prices are used to estimate the cost-efficient mitigation potential and its distribution across sectors and regions rather than a real-world policy^15^. We apply two global carbon price trajectories, US$150 and US$500 per tCO_2_e at 2070, consistent with earlier publications, as higher carbon prices cannot stimulate more technical options, but decrease consumption. In addition to CH_4_ emissions, N_2_O emissions from agricultural production are priced according to the GWP_100_ N_2_O price. In line with the focus of this study on non-CO_2_ emissions from agriculture, CO_2_ emissions from deforestation or other land-use change are not priced.

The second mitigation option is a shift towards consumption of diet containing less animal protein. The adoption of such a diet has been identified in the literature as a promising strategy to curb GHG emissions from the agriculture and global food systems^29,30^. This is in line with recommendations by the EAT-Lancet Commission, which proposes a healthier diet where whole grains, fruits, vegetables, nuts and legumes comprise a greater proportion of foods consumed. This diet includes calorie intake targets by food group and a total calorie intake target of 2,100 kcal (refs.^31,59–63^).

### Calculation of methane prices

The models are run for two options for the computation of the carbon price: MEF-ST (‘short-term’) and MEF-LT (‘long-term’). Both options use the same carbon price. We distinguish two price paths: US$150 and US$500 per tCO_2_e in 2005. Following Hotelling’s rule^58^, the optimal carbon price path follows a discount rate which is set to 5%. This results in the carbon price rates shown in Table 3.

We base our computation of the carbon price for CH_4_ on the GWP* equation in ref. ^11^, p. 3:

$${\text{GWP}}_{t}^{*}=\left(4\times E_{\text{SLCP}(t)}-3.75\times E_{\text{SLCP}(t-20)}\right)\times {\text{GWP}}_{100}$$

where ${\text{GWP}}_{t}^{*}$ measures the global warming potential of CH_4_ depending on emitted CH_4_ in year *t* and emitted CH_4_ 20 yr before (*t* - 20). Emitted CH_4_ in *t* appears a second time in the calculation of GWP* in *t* + 20:

$${\text{GWP}}_{t+20}^{*}=\left(4\times E_{\text{SLCP}(t+20)}-3.75\times E_{\text{SLCP}(t)}\right)\times {\text{GWP}}_{100}$$

Assuming a carbon price rate CPR_*t*_, a given CH_4_ emission *E*_SLCP(*t*)_ in year *t* is taxed twice and the total amount CP_*t*_ of that emission is given by:

$${\text{CP}}_{t}=\left(4\times E_{\text{SLCP}(t)}\times {\text{CPR}}_{t}-3.75\times E_{\text{SLCP}(t)}\times {\text{CPR}}_{t+20}\right)\times {\text{GWP}}_{100}$$

Since the path of the carbon price follows the discount rate, the carbon price rate in real prices remains the same in all years, CP_*t*+20_ = CP_*t*_.

The MEF-ST option of the carbon price disregards the flow term, that is, the price reward 20 yr after the emission occurred. Therefore, the carbon price of a CH_4_ emission in year *t* defined in US$ per tCH_4_ becomes:

$${\text{CP}}_{t}(\text{‘short-term’})=\left(4\times E_{\text{SLCP}(t)}\times {\text{CPR}}_{t}\right)\times {\text{GWP}}_{100}$$

The long-term option of the carbon price regards both terms, and the carbon price of a CH_4_ emission in year *t* defined as US$ per tCH_4_ is computed as:

$${\text{CP}}_{t}(\text{‘long-term’})=\left(0.25\times E_{\text{SLCP}(t)}\times {\text{CPR}}_{t}\right)\times {\text{GWP}}_{100}$$

Assuming GWP_100_ = 25 (AR4), the carbon prices for the two options are shown in Table 4. For comparison, the price rates based on GWP_100_ are also shown.

## Supplementary Material

Fig S1

Fig S2

Fig S3

Fig S4

S1

Reporting summary Further information on research design is available in the Nature Research Reporting Summary linked to this article.

## Figures and Tables

**Table 1:** Scenario matrix

Carbon pricing regime for CH_4_	No carbon price		Carbon price US$150 t^−1^		Carbon price US$500^t–1^	
	No dietary change	Low-animal-protein diet	No dietary change	Low-animal-protein diet	No dietary change	Low-animal-protein diet
No carbon pricing	BASE	BASE_D				
MEF-LT^a^			CP150_LT	CP150_LT_D	CP500_LT	CP500_LT_D
^GWP^100			CP150	CP150_D	CP500	CP500_D
MEF-ST^b^			CP150_ST	CP150_ST_D	CP500_ST	CP500_ST_D

a Methane equivalent factor long term (MEF-LT): 0.25 × 25 = 6.25.

b Methane equivalent factor short term (MEF-ST): 4 × 25 = 100. Note: the scenarios do not include residual climate change impacts on yields.

**Table 2:** Indicators for global agriculture by carbon pricing regime and carbon price level

result indicator	Carbon pricing regime	No carbon price	Carbon price US$150 t^−1^		Carbon price US$500 t^−1^	
		Low-animal-protein diet	No dietary change	Low-animal-protein diet	No dietary change	Low-animal- protein diet
**Added warming from CH_4_ emissions compared to 2010**	**No carbon pricing**	−36				
	**MEF-LT (= 6.25)**		−51	−80	−88	−109
	**GWP_100_ (= 25)**		−85	−107	−117	−131
	**MEF-ST (= 100)**		−115	−130	−132	−141
**Added warming from non-Co_2_ emissions compared to 2010**	**No carbon pricing**	−23				
	**MEF-LT (= 6.25)**		−34	−53	−58	−72
	**GWP_100_ (= 25)**		−53	−68	−74	−84
	**MEF-ST (= 100)**		−70	−81	−84	−91
**Total production index**	**No carbon pricing**	−13				
	**MEF-LT (= 6.25)**		−2	−15	−6	−17
	**GWP_100_ (= 25)**		−3	−15	−6	−17
	**MEF-ST (= 100)**		−4	−16	−8	−18
**Crop production index**	**No carbon pricing**	−8				
	**MEF-LT (= 6.25)**		−2	−10	−4	−11
	**GWP_100_ (= 25)**		−2	−9	−4	−11
	**MEF-ST (= 100)**		−2	−9	−4	−11
**Non-ruminant production index**	**No carbon pricing**	−30				
	**MEF-LT (= 6.25)**		−2	−30	−5	−31
	**GWP_100_ (= 25)**		−2	−30	−5	−30
	**MEF-ST (= 100)**		−1	−29	−4	−27
**ruminant production index**	**No carbon pricing**	−27				
	**MEF-LT (= 6.25)**		−8	−35	−18	−42
	**GWP_100_ (= 25)**		−14	−39	−25	−47
	**MEF-ST (= 100)**		−24	−46	−36	−52
**Producer price**	**No carbon pricing**	−16				
	**MEF-LT (= 6.25)**		5	−11	16	−2
	**GWP_100_ (= 25)**		8	−9	24	4
	**MEF-ST (= 100)**		17	−2	51	26

Average of models; percentage change relative to baseline in 2070.

**Table 3:** Carbon price rates (US$ per tCo_2_) by carbon price level and year

Carbon price level (US$ per tCo_2_e)	2030	2050	2070
150	23	62	165
500	78	207	549

**Table 4:** Carbon price rates for methane (US$ per tCH_4_) by carbon price level, pricing option and year

Carbon price level (US$ per tCo_2_e)	Pricing option	2030	2050	2070
150	GWP_100_	585	1,553	4,120
	MEF-ST	2,341	6,211	16,480
	MEF-LT	146	388	1,030
500	GWP_100_	1,951	5,176	13,733
	MEF-ST	7,803	20,704	54,934
	MEF-LT	488	1,294	3,433

## Data Availability

The data that support the findings of this study are available under the data portal of agro-economics modelling of the European Commission (https://datam.jrc.ec.europa.eu, climate change section). Source data are provided with this paper.

## Code availability

All programming codes are available from the corresponding author upon reasonable request.
