# Supplementary material for: Short- and long-term warming effects of methane may affect the cost-effectiveness of mitigation policies and benefits of low-meat diets
Source: Nat Food. Author manuscript; Available in PMC 2022 Feb 9. (PMC7612339; doi:10.1038/s43016-021-00385-8)
Supplement: S1 [file EMS141014-supplement-S1.pdf]

nature portfolio

https://doi.org/10.1038/s43016-021-00385-8

Supplementary information

Short- and long-term warming effects of methane may affect the cost-effectiveness of mitigation policies and benefits of low-meat diets

In the format provided by the authors and unedited

Short- and long-term warming effects of methane may affect the cost effectiveness of mitigation policies and benefits of low meat diets

Supplementary Information

Additional supporting Figures

Figure S1: ***Methane and nitrous oxide emissions for the baseline and 500 $/t carbon price scenarios****. World totals by year and model; (a) annual Mt CH_4; (b) annual Mt N_2O; (c) added warming for CH_4 emissions (°C); and (d) added warming for total non-CO_2 emissions °C).*

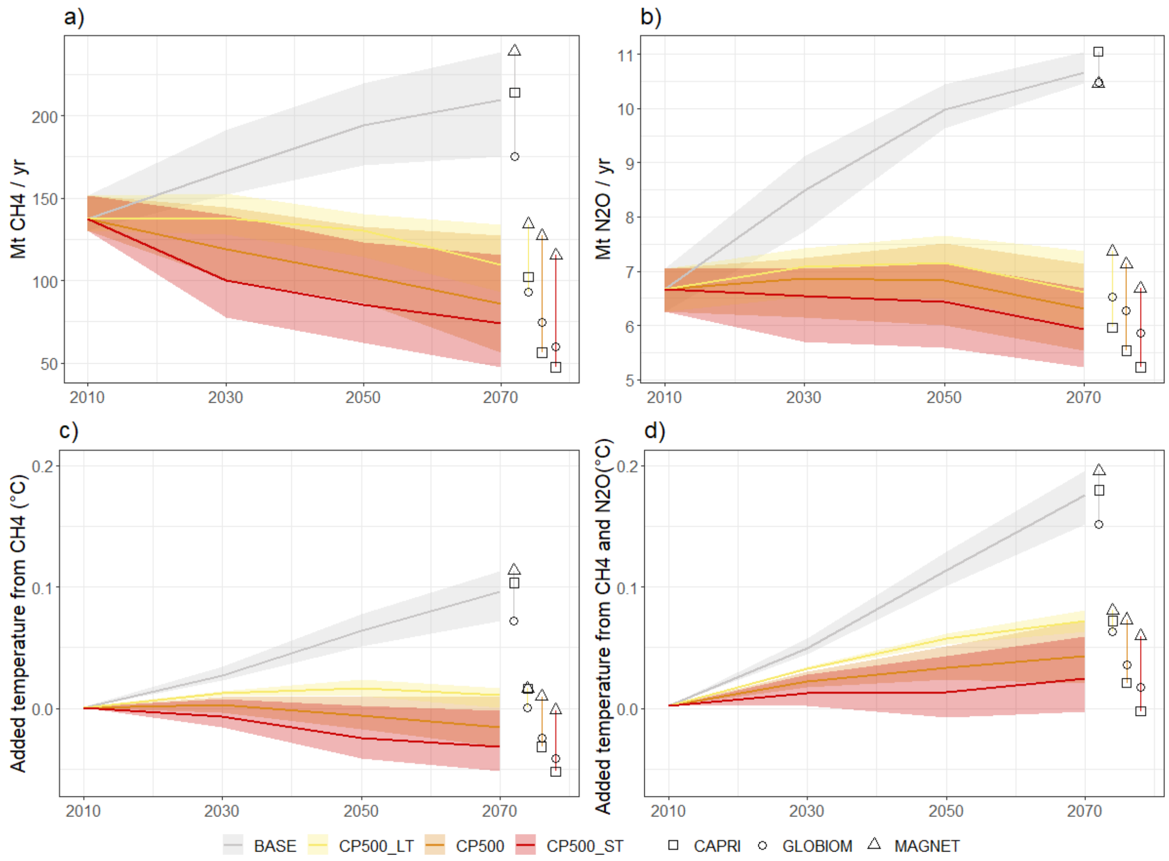

Note: The shading is the range (distribution across models for respective scenario) compared to the average (thick middle line)

Figure S2: ***Methane and nitrous oxide emissions for the baseline and short-term carbon price scenarios including dietary shifts.*** *World totals by year and model; (a) Annual Mt CH_4; (b) Annual Mt N_2O; (c) added warming for CH_4 emissions (℃); and (d) added warming for total non-CO_2 emissions (℃).*

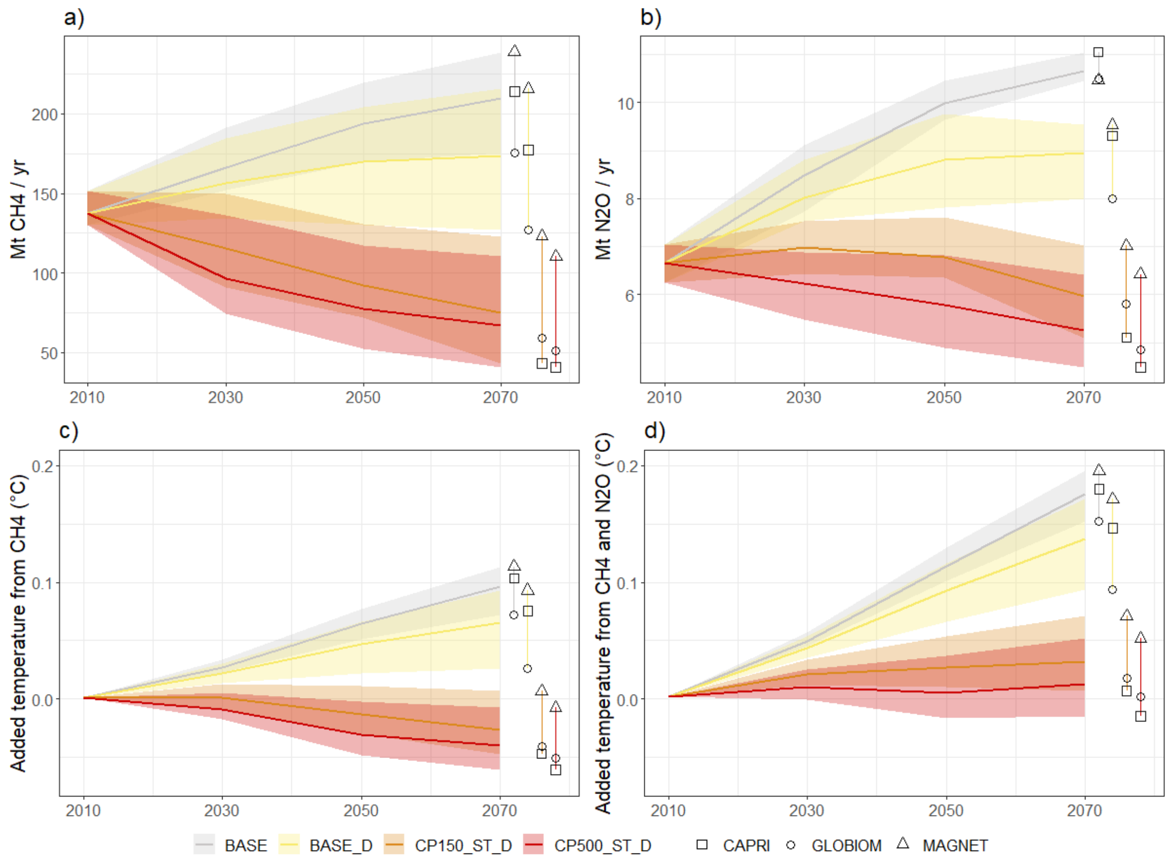

Note: The shading is the range (distribution across models for respective scenario) compared to the average (thick middle line)

Figure S3: ***Methane and nitrous oxide emissions for the baseline and long-term carbon price scenarios including dietary shifts.*** *World totals by year and model; (a) Annual Mt CH_4; (b) Annual Mt N_2O; (c) added warming for CH_4 emissions (℃); and (d) added warming for total non-CO_2 emissions (℃).*

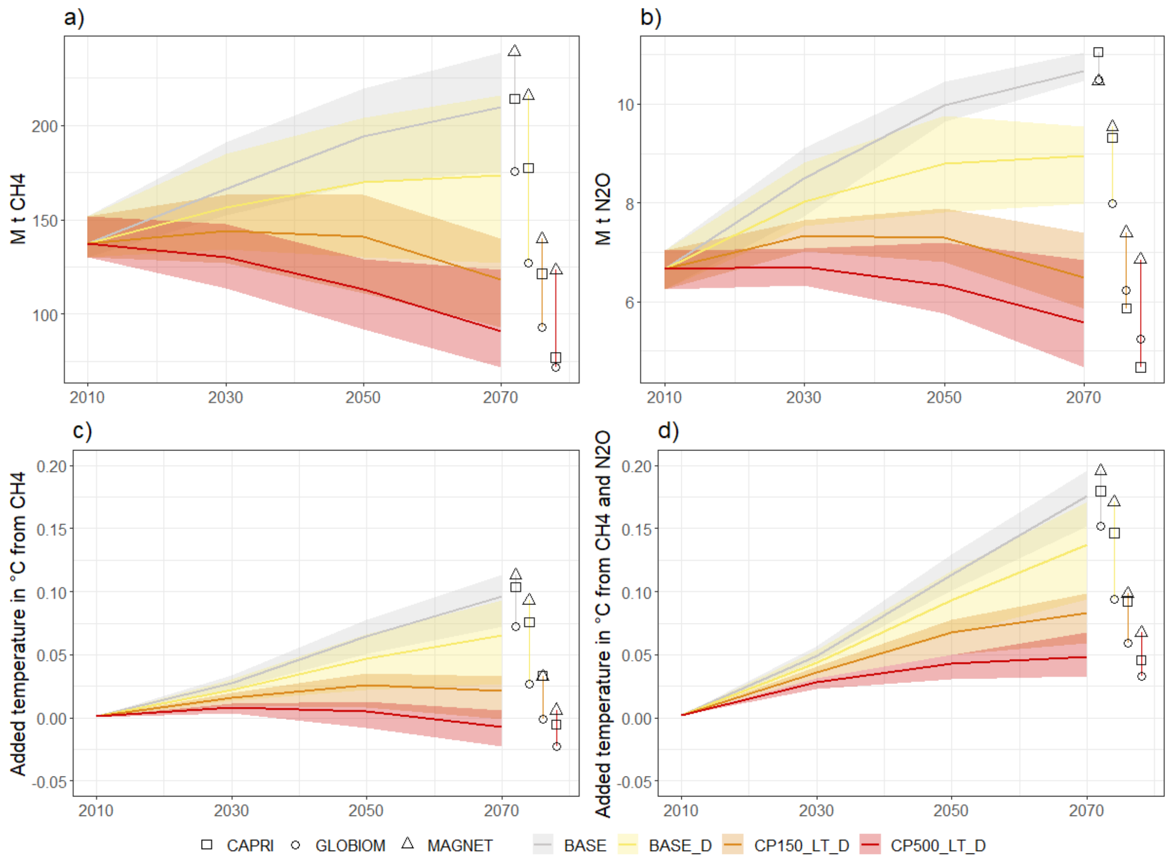

Note: The shading is the range (distribution across models for respective scenario) compared to the average (thick middle line)

Figure S4: ***Area, herd sizes, production and producer prices for crops, non-ruminants and ruminants aggregate products****.* *World totals for all scenarios, average model estimates, percentage change relative to the baseline: (a) non-dietary shift scenarios and (b) dietary shift scenarios*

(a)

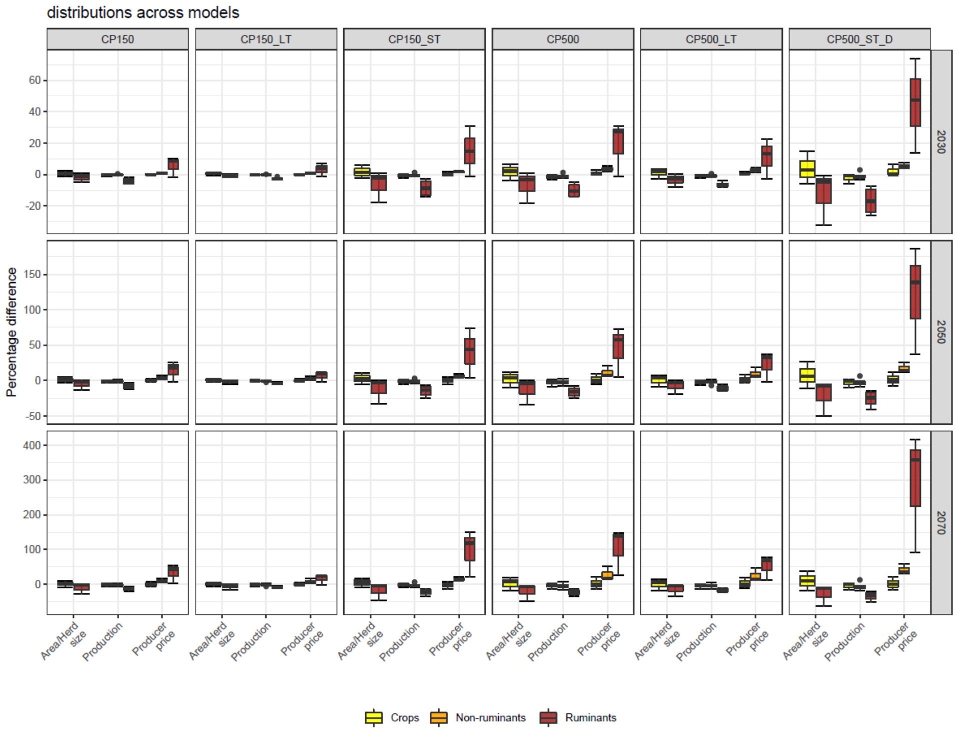

(b)

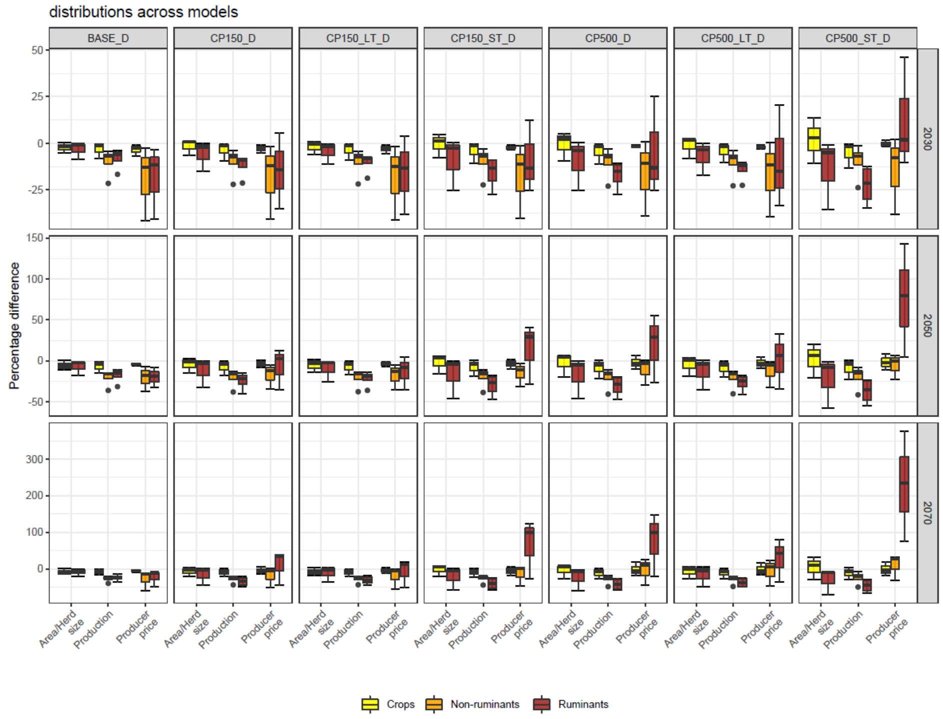

Note: non-ruminant herd sizes are not reported due to lack of detailed information by the models involved, which are relying on slaughtering statistics.
