# Supplementary material for: Short- and long-term warming effects of methane may affect the cost-effectiveness of mitigation policies and benefits of low-meat diets
Source: Nat Food. Author manuscript; Available in PMC 2022 Feb 9. (PMC7612339; doi:10.1038/s43016-021-00385-8)
Supplement: Reporting summary — Further information on research design is available in the Nature Research Reporting Summary linked to this article. [file EMS141014-supplement-Reporting_Summary.pdf]

nature research

Corresponding author(s): DBPR - NATFOOD-21021910-T

Last updated by author(s): Feb 19, 2021

Reporting Summary

Nature Research wishes to improve the reproducibility of the work that we publish. This form provides structure for consistency and transparency in reporting. For further information on Nature Research policies, see our Editorial Policies and the Editorial Policy Checklist.

Statistics

For all statistical analyses, confirm that the following items are present in the figure legend, table legend, main text, or Methods section.

n/a	Confirmed	
☒	☐	The exact sample size (*n*) for each experimental group/condition, given as a discrete number and unit of measurement
☒	☐	A statement on whether measurements were taken from distinct samples or whether the same sample was measured repeatedly
☒	☐	The statistical test(s) used AND whether they are one- or two-sided<br>*Only common tests should be described solely by name; describe more complex techniques in the Methods section.*
☒	☐	A description of all covariates tested
☒	☐	A description of any assumptions or corrections, such as tests of normality and adjustment for multiple comparisons
☒	☐	A full description of the statistical parameters including central tendency (e.g. means) or other basic estimates (e.g. regression coefficient) AND variation (e.g. standard deviation) or associated estimates of uncertainty (e.g. confidence intervals)
☒	☐	For null hypothesis testing, the test statistic (e.g. *F*, *t*, *r*) with confidence intervals, effect sizes, degrees of freedom and *P* value noted<br>*Give P values as exact values whenever suitable.*
☒	☐	For Bayesian analysis, information on the choice of priors and Markov chain Monte Carlo settings
☒	☐	For hierarchical and complex designs, identification of the appropriate level for tests and full reporting of outcomes
☒	☐	Estimates of effect sizes (e.g. Cohen's *d*, Pearson's *r*), indicating how they were calculated

Our web collection on statistics for biologists contains articles on many of the points above.

Software and code

Policy information about availability of computer code

Data collection	No specific software was used for data collection. The analysis relies on partly harmonized databases used by the three large-scale agri-economic models used in the study (CAPRI, GLOBIOM and MAGNET)
Data analysis	The software used for the analysis in the CAPRI and GLOBIOM models is GAMS (www.gams.com). In the case of the MAGNET model, GEMPACK (https://www.copsmodels.com/gempack.htm) was used as the main software of analysis. Most of the reporting was done by using the open-source software R (www.r-project.org).

For manuscripts utilizing custom algorithms or software that are central to the research but not yet described in published literature, software must be made available to editors and reviewers. We strongly encourage code deposition in a community repository (e.g. GitHub). See the Nature Research guidelines for submitting code & software for further information.

Data

Policy information about availability of data

All manuscripts must include a data availability statement. This statement should provide the following information, where applicable:

- Accession codes, unique identifiers, or web links for publicly available datasets
- A list of figures that have associated raw data
- A description of any restrictions on data availability

The data that support the findings of this study are available under the Data portal of agro-economics Modelling of the European Commission (https://datam.jrc.ec.europa.eu/datam/public/pages/index.xhtml).

Field-specific reporting

Please select the one below that is the best fit for your research. If you are not sure, read the appropriate sections before making your selection.

☐ Life sciences ☒ Behavioural & social sciences ☐ Ecological, evolutionary & environmental sciences

For a reference copy of the document with all sections, see nature.com/documents/nr-reporting-summary-flat.pdf

Behavioural & social sciences study design

All studies must disclose on these points even when the disclosure is negative.

Study description	Methane has been wrongly considered as a long-lived pollutant in economic studies and policy analyses for more than a decade. This study is to our knowledge the first one integrating and quantifying the short-lived nature of methane in agricultural emission mitigation policies. This has important implications for food markets, in case the agricultural sector needs to be further decarbonized, and stresses the role of low-meat diets in future climate policies. Our work has important implications for the appropriate use of metrics measuring global warming
Research sample	We use carbon price pathways computed by the Integrated Assessment Modeling Consortium (ehttps://www.iamconsortium.org/) and consistent with different levels of climate mitigation ambition during the present century. These carbon prices are used as proxies for mitigation efforts at a global scale and applied to the agricultural sector by means of the CAPRI, GLOBIOM and MAGNET large-scale agro-economic models.
Sampling strategy	No sampling is needed for this study, scenarios were designed and participation was open to most global large-scale agricultural economic models performing climate mitigation and adaptation work
Data collection	Updated and harmonized databases of the three mentioned models are used, no further data collection efforts were needed for this study
Timing	Our analysis is ex-ante and goes until 2070
Data exclusions	No data were excluded from the analysis
Non-participation	The MAGPIE (https://www.pik-potsdam.de/en/institute/departments/activities/land-use-modelling/magpie) and AIM (https://www-iam.nies.go.jp/aim/) models were not included in this paper due to lack of time parameterizing the model for the presented analysis. They have access to our scenario protocols and their participation could be re-considered during the review process, provided the editor and reviewers find this beneficial and appropriate
Randomization	Participants in the study (i.e. models) were not allocated into experimental groups

Reporting for specific materials, systems and methods

We require information from authors about some types of materials, experimental systems and methods used in many studies. Here, indicate whether each material, system or method listed is relevant to your study. If you are not sure if a list item applies to your research, read the appropriate section before selecting a response.

Materials & experimental systems

n/a	Involved in the study	
☒	☐	Antibodies
☒	☐	Eukaryotic cell lines
☒	☐	Palaeontology and archaeology
☒	☐	Animals and other organisms
☒	☐	Human research participants
☒	☐	Clinical data
☒	☐	Dual use research of concern

Methods

n/a	Involved in the study	
☒	☐	ChIP-seq
☒	☐	Flow cytometry
☒	☐	MRI-based neuroimaging

April 2020
